# Oxygen Toxicity to the Immature Lung—Part I: Pathomechanistic Understanding and Preclinical Perspectives

**DOI:** 10.3390/ijms222011006

**Published:** 2021-10-12

**Authors:** Yesi Choi, Lisa Rekers, Ying Dong, Lena Holzfurtner, Maurizio J. Goetz, Tayyab Shahzad, Klaus-Peter Zimmer, Judith Behnke, Jonas Behnke, Saverio Bellusci, Harald Ehrhardt

**Affiliations:** 1 Department of General Pediatrics and Neonatology, Justus-Liebig-University, Universities of Giessen and Marburg Lung Center (UGMLC), 35392 Giessen, Germany; Yesi.Choi@med.uni-giessen.de (Y.C.); Lisa.Rekers@med.uni-giessen.de (L.R.); Ying.Dong@paediat.med.uni-giessen.de (Y.D.); Lena.Holzfurtner@med.uni-giessen.de (L.H.); maurizio.j.goetz@med.uni-giessen.de (M.J.G.); tayyab.shahzad@paediat.med.uni-giessen.de (T.S.); klaus-peter.zimmer@paediat.med.uni-giessen.de (K.-P.Z.); judith.behnke@paediat.med.uni-giessen.de (J.B.); 2 Department of Internal Medicine II, Universities of Giessen and Marburg Lung Center (UGMLC), Cardiopulmonary Institute (CPI), 35392 Giessen, Germany; saverio.bellusci@innere.med.uni-giessen.de; 3 Department of Internal Medicine V, Justus-Liebig-University, Universities of Giessen and Marburg Lung Center (UGMLC), 35392 Giessen, Germany; jonas.behnke@innere.med.uni-giessen.de

**Keywords:** chronic lung disease, bronchopulmonary dysplasia, preterm, reactive oxygen species, inflammation, lung injury, rodent, therapeutic approach

## Abstract

In utero, the fetus and its lungs develop in a hypoxic environment, where HIF-1α and VEGFA signaling constitute major determinants of further development. Disruption of this homeostasis after preterm delivery and extrauterine exposure to high fractions of oxygen are among the key events leading to bronchopulmonary dysplasia (BPD). Reactive oxygen species (ROS) production constitutes the initial driver of pulmonary inflammation and cell death, altered gene expression, and vasoconstriction, leading to the distortion of further lung development. From preclinical studies mainly performed on rodents over the past two decades, the deleterious effects of oxygen toxicity and the injurious insults and downstream cascades arising from ROS production are well recognized. This article provides a concise overview of disease drivers and different therapeutic approaches that have been successfully tested within experimental models. Despite current studies, clinical researchers are still faced with an unmet clinical need, and many of these strategies have not proven to be equally effective in clinical trials. In light of this challenge, adapting experimental models to the complexity of the clinical situation and pursuing new directions constitute appropriate actions to overcome this dilemma. Our review intends to stimulate research activities towards the understanding of an important issue of immature lung injury.

## 1. Introduction

The fetus and its lungs develop within a hypoxic environment. In utero, oxygen tensions do not exceed 25–30 mmHg, which is a prerequisite for physiological function—especially of the hypoxia-inducible factor (HIF) family of transcription factors that not only control the metabolic situation, but also constitute key drivers of angiogenesis in the developing lungs [1]. The HIF family regulates more than 2000 genes, mainly involved in tissue growth and homeostasis. Within this family, HIF-1α is the best studied representative, which primarily directs vascular development via vascular endothelial growth factor (VEGF)-A, angiopoietins, and oxygen consumption [2,3]. Thereby, the induced gene regulations by HIF-1α direct both tissue oxygen delivery and oxygen consumption. With preterm, delivery the fetus is exposed to 4–5-fold higher tensions of oxygen measured in the arterial blood, and the local oxygen concentrations applied to the lungs to meet oxygen saturation targets depend on the capacities for gas exchange [1]. From the available experimental evidence, it becomes clear that brief changes in oxygen tension can have tremendous effects on the balanced in utero situation that extend far beyond the neonatal period [4]. While the HIF family is well suited to counteracting hypoxemic episodes, nature is not prepared to counteract hyperoxic events until shortly before birth. Shortly before birth, antioxidative defense mechanisms are established through the fetus’ own synthesis and placental transfer.

Two pioneering studies in rodents displayed the wide range of genes being regulated when animals were exposed to hyperoxia directly after birth, which included central pathways of lung development such as transforming growth factor (TGF)-β and cell cycle control. A recent study demonstrated that even short-term exposure of newborn mice to oxygen for 30 min induced subtle but persistent typical changes in lung structures related to secondary crest formation, which is the critical step of lung development at this stage [4]. In another study using an identical model, it was demonstrated that even lower fractions of oxygen (40% and 65%) than the 80% usually applied in rodent models caused a persistent increase in lung oxidative stress response and immune cell reactivity in the bronchoalveolar lavage that was mainly constituted by macrophages. These data point towards a persistent inflammation in the lungs after hyperoxic exposure in the neonatal period [5]. It should be noted that hyperoxia with intermittent hypoxia affected the number and extent of gene regulations in an unfavorable direction [6,7]. In one study in rats, only the combination of hyperoxia with hypoxemic episodes decreased the antioxidative defense mechanisms, as well as HIF-1α and its downstream activity [8]. These data are not surprising, since hypoxemia leads to the accumulation of purine derivatives such as hypoxanthine during hypoxia–reoxygenation injury. During reoxygenation, these derivatives are metabolized to superoxide anions, which provoke oxidative stress response and lung tissue damage that are boosted by further mechanisms of oxidative bursts, including NADPH oxidase activation [9,10]. These alterations are not restricted to genes involved in antioxidant defense, DNA repair, organ development, and growth, but also include the induction of inflammatory genes. These pathomechanistic insights are consistent with previous studies, mainly conducted in rodents, with prevailing results that hyperoxia and hypoxia both impaired lung development. This was attributed to an overexpression of TGF-β signaling, while HIF-1α and VEGFA signaling were dysregulated [11]. This critical function of pulmonary vessel formation and growth for total lung development was further confirmed by a fundamental study that proved a direct link between TGF-β action and distorted VEGFA function and lung development by hyperoxia [12]. The deleterious effects of oxygen are aggravated by secondary impacts such as pre- and perinatal infections that exert their injurious actions via identical pathways. It remains to be determined whether oxidative lung damage differs between gestational ages at birth and during the longitudinal course of treatment in the NICU.

## 2. The Link between Oxygen Exposure, ROS Production and BPD

During the transition from the intrauterine to extrauterine environment, preterm infants are challenged by an abrupt change in oxygen tensions and oxygen needs. These are determined by the oxygen targets required to ensure their survival. Currently, narrow oxygen saturation targets represent the gold standard of care. However, controversies remain with regard to the strategy of optimal oxygen targeting. While higher concentrations improve survival rates but increase the risk of neonatal morbidity, lower fractions reduce the risk of severe morbidities but enhance mortality [2]. The targets might be revised within the next several years based on new clinical outcome data. Nevertheless, the dramatic change in oxygen tensions after birth will remain. Basic researchers and clinicians continue to be faced with this challenge, and efficient strategies to prevent oxygen toxicity to the preterm infant that go beyond antenatal steroid exposure and surfactant application are urgently required, since both sustainably reduce the fraction of inspired oxygen after birth, but fail to reduce the required tissue oxygen tensions. Our review summarizes the experimental evidence on this topic, promising therapeutic approaches, and the disparities between preclinical rodent studies and the human situation that may account for the difference in their effectiveness. Experimental studies have detailed the lung pathologies induced by reactive oxygen species (ROS), but mechanistic links remain limited.

Oxygen is the source of life, and is physiologically reduced to water, which is mainly executed within several steps in the mitochondria. Physiological ROS levels are a prerequisite for redox-sensitive signal transduction, and play important roles in development and tissue homeostasis, but overwhelming ROS production puts the immature lungs at risk of distortion of further developmental steps. Due to the immaturity of the system, reduction of oxygen intermediates such as superoxides, hydrogen peroxide, and hydroxyls is limited, and they can exert their toxic and deleterious effects on cell membranes, enzymes, proteins, and DNA [2]. Mitochondria play a central role, but further relevant sources of ROS arise from other intracellular compartments, including plasma membranes, peroxisomes, and the endoplasmic reticulum. In addition, free circulating transition metals—including iron and the ROS-producing enzymes such as peroxidases, NADPH, xanthine oxidases, lipoxygenases, myeloperoxidase, nitric oxide synthase, and cyclooxygenases—contribute to the excess of ROS during hyperoxia [13]. Mitochondria, again, are recipients of ROS products, and their key function is ROS reduction, but they are prone to oxidative self-injury. The capacity for ROS production is elevated in newborn infants, since their higher levels of free iron compared to older infants and adults boost the Fenton reaction that leads to the production of highly toxic hydroxyl radicals during ROS generation [14]. Both immaturity and overload of the mitochondrial ROS reduction system and mitochondrial damage per se have been identified as key features in the pathogenesis of BPD. Even short periods of exposure to higher oxygen tensions and increase in ROS production boost HIF-1α ubiquitination and its proteasomal degradation [2]. A study of glutaredoxin 1 (Grx1)-knockout mice confirmed the important roles of HIF-1α and nuclear factor (NF)-κB during hyperoxia and lung injury. Grx1 is a thiol transferase, and its main function is to decrease glutathione–protein adduct levels. In these knockout mice, HIF-1α levels were increased, with concomitant upregulation of VEGFA and VEGF receptor 2, while excess NF-κB and inflammation were prohibited, resulting in better preserved alveolar and vascular structures [15]. These data provide evidence that the preservation of HIF-1α function has the potential to prevent the deleterious effects of inflammation on lung development. 

Efficient ROS detoxification is essential in preventing situations of oxidative injury. The family of SOD enzymes is quickly overwhelmed when high amounts of ROS are released, while the reserve of SOD is too limited in the immature lung to counteract the effects of ROS. SOD-1 in the cytoplasm, SOD-2 located in the mitochondria, and extracellular SOD-3 constitute the key enzymes responsible for the dismutation of superoxides, and are supported by the downstream action of oxidoreductases, catalases, reductases, and peroxidases.

Further dimensions of complexity arise from the clinical situation. Most preterm infants experience restrictions in nutritional supply, abnormalities in lung development, or inflammatory injury prior to birth, which significantly impact on their lung status and defense mechanisms. While in animal experiments hyperoxia to rodents is mostly restricted to several days, with a continuum of oxygen supply within the late saccular and early alveolar stages of lung development, preterm infants are exposed to varying oxygen concentrations depending on the severity of gas exchange restrictions for weeks to months, along with short-term fluctuations in oxygen supply depending on the respiratory status. The inflammatory response leading to lung injury is thereby not solely determined by ROS production due to oxygen exposure, but by the extent of prenatal inflammation provoked by infection and suppression of the inflammatory response by prenatal corticosteroid administration.

A further dimension that deserves more detailed clarification is the microbial colonization in utero and after birth. Clear associations have been demonstrated with the extent of lung injury, but the few preclinical studies so far do not deliver congruent results [16,17]. Therefore, this review is restricted to the available research data, mostly on isolated ROS toxicity, but important studies combining hyperoxic and infectious injury are cited where appropriate. Recently, several studies have revealed that the concentration of oxygen provided significantly impacts lung function readouts and the histological picture of lung injury. Due to the paucity of preclinical research data on lower concentrations of oxygen, and our focus on ROS mediated lung injury, we mostly report data on high concentrations of oxygen, between 60 and 90%.

## 3. The Pathomechanisms of ROS Injury to the Immature Lung

**Figure 1:**
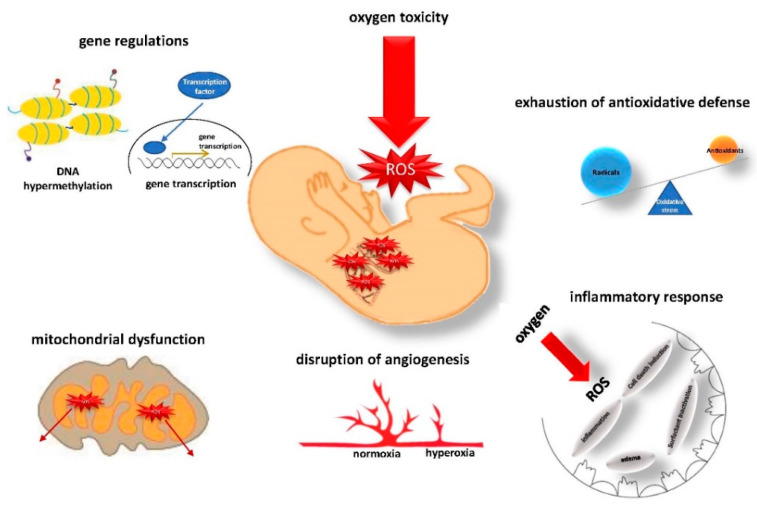
ROS production and downstream pathologies in the immature lung. After birth, the preterm infant and its lungs are exposed to a relatively hyperoxic environment compared to the intrauterine situation. ROS induce alterations in gene regulation and mitochondrial function, along with disruption of further pulmonary vasculogenesis and an inflammatory response causing damage to the immature lung. The excess ROS production is aggravated by restricted antioxidative defense mechanisms, resulting in acute and long-term injuries and insults to the lung and further lung development.

The deleterious actions of ROS on the immature lung can be separated into different categories, and comprehensive understanding of their pathomechanistic roles is a prerequisite to guiding novel therapeutic approaches (summarized in Figure 1).

### 3.1. Gene Regulation and Epigenetic Alterations

Several studies have demonstrated the extensive effects of hyperoxia and ROS on gene regulation. Similarly, the hyperoxia rodent models demonstrated prevailing results of dramatic changes in gene methylation status and transcriptome regulation, affecting more than 1000 genes. Within the dominant pathways identified, immune-system-related genes and inflammatory responses were identified as main targets [18]. Studies in newborn rats subjected to hyperoxia displayed DNA methylation in genes involved in hyperoxia, which mediated the alteration of alveolarization and signaling receptors and their proteins involved in lung growth and differentiation [19]. The most prominent candidates identified include factors involved in TGF-β signaling with key genes—such as Tgfbr1, Crebbp, and Creb1—that constitute a key developmental and injurious pathway to the lungs [20]. As these effects continue after the end of hyperoxia treatment, mostly with hypermethylation patterns, a persisting change in lung phenotype due to DNA methylation is suggested [20].

Within the antioxidative defense mechanisms, the transcription factor Nrf2 determines the resting state and induced expression of antioxidant and cytoprotective genes upon oxygen sensing. Nrf2 is rapidly induced upon hyperoxic exposure. Among its multiple functions, Nrf2 is involved in stem cell function, autophagy, metabolism, and protein function. It becomes clear that Nrf2 plays a central role within the complex network of antioxidative and cytoprotective regulations that are activated by oxygen sensing. Key functions of Nrf2 include GSH synthesis, NADPH production and regeneration, ROS detoxification, activation of antioxidant systems—including Txn production—and heme and iron metabolism [21]. In contrast, its genetic deletion aggravates lung injury and causes further distortion of lung development by hyperoxia [22].

### 3.2. Antioxidative Defense and Mitochondrial Dysfunction

Hyperoxia leads to mitochondrial stress and dysfunction, along with the accumulation of ROS metabolites and damage to the immature lung—particularly in the alveolar epithelium [23,24]. The mechanistic link was documented on a molecular level by overexpression of SOD2 that markedly reduced alveolar epithelial damage [25]. Moreover, the lung-tissue-specific overexpression of SOD3 better preserved alveolar structure formation during hyperoxia [26]. Similarly, the transient overexpression of SOD3 via inhalation before hyperoxia exposure maintained nitric oxide (NO) bioavailability and subsequent cGMP activity, while the pro-inflammatory activation of NF-κB was prohibited [27]. These data provide further proof of the connection between ROS production and activation of the inflammatory response. The increase in ROS in the lung tissue is the initial key step that provokes the inflammatory response in the immature lung, with the release of pro-inflammatory cytokines and the attraction of the pro-inflammatory leukocytes. Treatment with the mitochondria-targeted antioxidant mitoTEMPO 2-(2,2,6,6-tetramethylpiperidin-1-oxyl-4-ylamino)-2-oxoethyl) triphenyl-phosphonium chloride prevented distortion of alveolarization and right ventricular hypertrophy, suggesting that all typical BPD features were successfully prevented. Furthermore, the inhibition of mitochondrial ROS production abrogated the secondary effects of ROS damage, including the activation of the NOX1 gene, while other isoforms such as NOX2 and NOX4 were not regulated, confirming the specificity towards NOX1 [28].

The cytochrome P450 family contains the monooxygenases CYP1A1 and CYP1A2, which were identified as playing important roles in reducing injury in adult models of hyperoxic lung injury. Unsurprisingly, the induction of CYP1A1 function by β-naphthoflavone reduced the classical features of hyperoxic lung injury in mice, as did the transgenic overexpression of the CYP1A1 promoter in newborn mice subjected to hyperoxia. The studies on the CYP1A1 promoter overexpression found that the hyperoxic exposure per se induces the activation of the promoter and the CYP1A1 activity that is responsible for the attenuation of hyperoxic lung injury [29,30]. Further studies extending these initial findings have provided evidence that β-naphthoflavone action is not restricted to CYP1A1, as postnatal administration in CYP1A1-knockout mice induced the expression of CYP1A2 and NAD(P)H quinone oxidoreductase, which led to comparable reduction in lung injury to that in wild-type mice [31]. In contrast to the expectations, the severity of lung injury was not augmented in CYP1A2-knockout mice, suggesting a selective protective role for CYP1A1, but not CYP1A2. Again, the application of β-naphthoflavone in CYP1A2-knockout mice revealed that its action is mainly mediated by the NAD(P)H quinone oxidoreductase [32]. These data constitute further documentation of important differences in biological pathways between newborns and adults, which account for the disparities in hyperoxic lung injury. The ROS detoxification systems display further redundancies of gene regulation, as described for the glutathione peroxidase system in the rodent model of neonatal hyperoxic lung injury [33]. Gender-specific evaluation of CYP1A1 and CYP1A2 activation after hyperoxia exposure revealed augmented activation in female mice, which might contribute to the gender-specific disparities in the extent of lung injury, to the disadvantage of the male gender [34].

In addition to direct targeting of the CYP1A family, plenty of further strategies have been pursued aiming to reduce ROS production. Within the chain of oxygen radical production, the Fenton reaction plays a central role, converting hydrogen peroxide into hydroxyl radicals that are catalyzed by bivalent iron. Aerosolized delivery or intraperitoneal injection of deferoxamine during and after the hyperoxic injury reduced the severity of lung injury. It should be noted that both alveologenesis and pulmonary vessel formation with improved HIF-1α and VEGFA signaling were better preserved in deferoxamine-treated animals [35,36].

Nrf2 is the key activator of the antioxidant stress response that includes NAD(P)H quinone oxidoreductase. Therefore, its dominant antioxidative role—which was demonstrated in NAD(P)H-quinone-oxidoreductase-knockout mice, and by the sustainable upregulation of β-naphthoflavone that prevented the features of hyperoxic lung injury—is not surprising [32,37].

### 3.3. Disruption of Angiogenesis

As detailed previously, the hyperoxia-induced dysregulation of HIF family member function constitutes a hallmark that causes disturbance of further angiogenesis. Although NO is mostly applied in clinics due to its acute pulmonary arterial antihypertensive and vasodilative action, its lung-vessel-growth-promoting activities are of major importance, as documented in several animal models. NO is the downstream executioner in the cascade of HIF and VEGFA, and is suppressed during ROS injury. ROS impairs NO signaling and stimulates smooth muscle cell growth and alterations, as well as pulmonary vascular remodeling. Studies using inhaled NO or, alternatively, systemic administration of NO donors such as L-citrulline, have proven efficient to preserve lung development under hyperoxic conditions [38]. The beneficial effects of NO were ascribed to its downstream mediator function of VEGFA but, simultaneously, an increase in VEGFA was detected, suggesting a positive feedback amplification of action [39]. Even the retarded application of inhaled NO after hyperoxic exposure proved efficient to promote distal lung growth, resulting in lung catch-up growth [40]. Studies in lambs allowed sophisticated analyses of pulmonary function, demonstrating that mitochondrial dysfunction and oxidative stress lead to vascular maldevelopment and pulmonary hypertension. Disease pathology was ascribed to distorted NO function and signal transduction, along with reduced soluble guanylate cyclase and cGMP levels, prompting constriction of pulmonary vessels [41,42]. Further studies using leukotriene inhibition as another approach in this context provided comparable results [43]. The anti-inflammatory role of NO in the prevention of inflammation and lung injury by hyperoxia was documented in a newborn NO inhalation model, where inflammatory genes such CCXL1 or IL6 were downregulated, the influx of inflammatory leukocytes was attenuated, and alveolar fibrin deposition and septum thickness were reduced [27]. In summary, NO displays potent pro-angiogenetic and anti-inflammatory properties.

### 3.4. Inflammatory Response

ROS stimulate a pro-inflammatory response in the immature lung, with an overshoot of classical cytokines such as IL-1β and TNF-α, excess NF-κB activation, and an influx of inflammatory macrophages and neutrophils [44]. Of particular importance, these secondary changes induce direct toxic injury to the alveolar structures and surfactant function, and highly impede further development of alveolar and vascular development and lung mesenchymal stem cell function [44,45,46,47]. Polymorphonuclear leukocytes (PMNs) likely constitute a major source of ROS, perpetuating and amplifying the initial pathomechanistic changes provoked by oxygen toxicity. Their particular tissue-damaging function has been ascribed to the simultaneous release of a variety of proteases that aggravate tissue injury and maintain and boost the release of pro-inflammatory cytokines and the activation of pro-inflammatory pathways, with the NF-κB pathway constituting the key pathway [44]. Activation of several further pathways has been described in this context, with TGF-β and the WNT/b-catenin system constituting two further pathways of lung injury. Their function during lung injury was documented by the treatment with rosiglitazone in particular [48]. 

**Figure 2:**
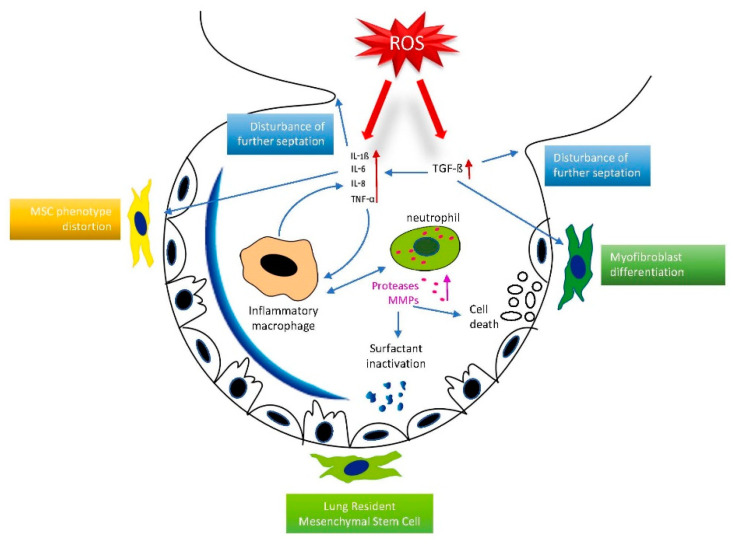
ROS-mediated pulmonary inflammatory response in the immature lung. Reactive oxygen species induce a pulmonary inflammatory response via overexpression of pro-inflammatory cytokines and attraction of inflammatory macrophages and neutrophils. Surfactant inactivation, cell death induction of lung cells, lung-resident mesenchymal stem cell phenotype distortion, and rarefication of septation constitute the hallmarks of BPD’s pathology.

TGF-β constitutes an important pathway of lung injury aggravation because of its stimulation of the inflammatory response in the lungs, as well as cell death. However, the attenuation of NF-κB signaling leads to further excess in TGF-β activation and disruption of angiogenesis, which was attributed to VEGF receptor 2 regulation [49,50]. It should be noted that hyperoxia and hypoxia both lead to the upregulation of TGF-β and WNT/b-catenin signaling, along with inflammation and increased lung injury. The key events leading to ROS-induced inflammatory injury are summarized in Figure 2.

One of the most recently studied events in the pulmonary pro-inflammatory response in BPD is the formation of the inflammasome. This complex is a key upstream executor that initiates the hyperoxia-induced injury; it is assembled from multiple proteins located in the cytoplasm, and is composed of sensor and adaptor proteins and inflammatory caspases, with caspase-1 performing the decisive function. It is activated in response to endogenous and exogenous stimuli, including ROS or LPS. The main action of inflammasome activation is the maturation and release of pro-inflammatory cytokines, with IL-1β constituting the most decisive in the context of BPD, along with the activation of caspases such as caspase-8, with cell death induction as the key effector function [51]. Treatment of mice exposed to hyperoxia with the irreversible caspase-1 inhibitor Ac-YVAD-CMK markedly reduced lung injury, with improved alveolar and vascular development, along with reduced IL-1β production [52].

Despite the initiating and decisive role of ROS in causing lung damage, thus far, no convincing therapeutic approaches have been identified for clinical evaluation. Therefore, therapeutic approaches focus more on the downstream actions of inflammation and the distortion of further lung development. A recent review on this topic has compiled the tremendous improvements in the understanding of normal and pathological alveolarization, which cannot be covered within the present review in such detail [53].

## 4. Preclinical Strategies to Prevent ROS-Induced Lung Injury

**Figure 3:**
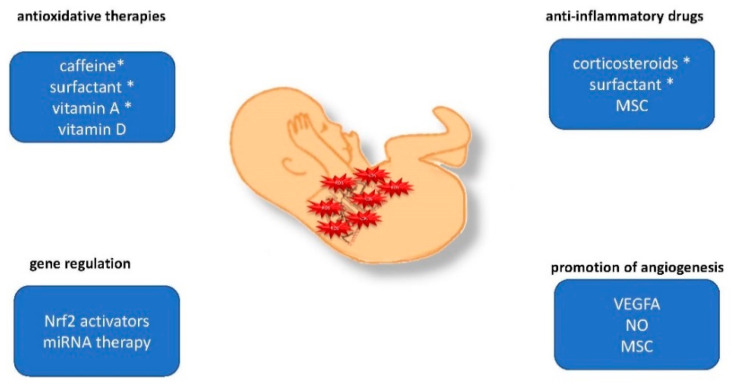
Therapeutic approaches to counteract the overexpression of ROS activity and its downstream actions in the immature lung. Preclinical studies in rodents identified several highly promising strategies to counteract ROS production and the activation of downstream injurious actions in the immature lung. Strategies are categorized by their main documented or postulated mode of action. * Therapeutic approaches with proven efficacy in the preterm infant to prevent or treat BPD.

Therapeutic approaches focused on the aforementioned areas of ROS action are still mainly restricted to preclinical research in rodent models of hyperoxic lung injury. Therefore, we will now summarize the most recent experimental insights that have the potential to guide further clinical strategies (Figure 3). The therapeutics with already proven efficacy are detailed in the second chapter of our review, and are detailed within several tables for their mode of action [54].

### 4.1. Gene Regulation and Epigenetic Alterations

Several approaches have tested the potential of augmenting Nrf2 activity, including preconditioning of the lung by targeting Keap1 and treatment with rapamycin, which resulted in increased baseline Nrf2 levels and antioxidant capacity. While the inflammatory response to hyperoxia was not altered in Nrf2-augmented mice, hypoalveolarization was markedly reduced [4,55]. In recent years, it has become clear that miRNA regulations constitute key events in the pathogenesis of BPD in hyperoxic injury models, and comparable changes have been documented in infants with BPD [56]. One recent study on miRNA 29b using the combined model of intrauterine inflammation induced by LPS and postnatal hyperoxia provided a link between miRNA regulation and histone methylation patterns. While exposure to hyperoxia downregulated both miRNA 29b levels and histone 3 and 4 methylation patterns, nanoparticle delivery of miR-29b on day 3 reverted these changes and partially reversed lung histopathology, with reduced septal wall thickness but unchanged alveolar air space [57]. One further dimension arises from the observed specific epigenetic regulation of genes implicated in cell cycle control, pulmonary vessel formation, vascular remodeling, and mesenchymal stem cell function in female mice, while in male mice, endothelium developmental pathways were specifically altered [58].

### 4.2. Antioxidative Defense

Tetrandrine is a potent suppressor of oxidative stress and inflammation; its application increased levels of antioxidant enzymes that were accompanied by a reduction in NF-κB activation, cellular lung inflammation, apoptosis, and fibrosis in rats exposed to hyperoxia [59]. The oxidoreductase thioredoxin reductase-1 reduces oxidized thioredoxin-1; its inhibition by aurothioglucose in newborn mice exposed to hyperoxia promoted Nrf2-related gene expression and lung development that was related to increased antioxidative activity [60]. Lipoxin A4 reduced both the oxidative stress response and the inflammatory response in the lung, which was followed by improved lung histopathology and function [61].

Hydrogen gas and hydrogen-rich water are highly potent eliminators of highly active ROS. Although not studied in the hyperoxic lung injury model, in the LPS rat model of BPD, the comparable mechanisms suggest that it is sufficiently potent to reduce the oxidative stress and protein oxidation in the immature lung [62]. Postnatal treatment of newborn mice exposed to hyperoxia with quercetin—a potent antioxidant and radical scavenger—potently reduced lung injury together with the features of lung inflammation, including the influx of inflammatory cells and lung tissue NF-κB activation. The key action of quercetin was ascribed to the upregulation of CYP1A1, CYP1B1, and NQO1 mRNA and protein levels, all of which have potent antioxidative properties [63].

The deficiency of vitamin D has been repeatedly associated with deregulation of redox cell signaling pathways. Several independent studies in newborn rodents exposed to hyperoxia provide convincing evidence that vitamin D preserves lung histology in hyperoxia-exposed animals. All such studies have in common the fact that they demonstrated reduced cell death induction in the lungs, but the mechanism of vitamin D has not been fully elucidated so far. Attenuation of downstream inflammatory activation of ROS—including toll-like receptor 4 and NF-κB signaling—together with decreased pro-inflammatory cytokine levels of IL-1β and TNF-α, among others, under vitamin D therapy, point towards a potential role of vitamin D in ROS detoxification, of which experimental evidence is urgently needed [64,65]. Vitamin A is another candidate with antioxidative properties. While it was originally recognized for its lung-growth-promoting function in the developmental stages of prematurity, its antioxidative capacities were largely neglected. Thereby, the combination of vitamin A plus retinoic acid (its active metabolite) seems more promising to refill lung tissue retinoid stores in rodents, and this approach proved efficient to reduce protein oxidation and DNA damage in the lungs of newborn mice exposed to hyperoxia, and to attenuate Nrf2 activation and the structural and functional changes of hyperoxic lung injury [66]. In contrast, the approach of antioxidative therapy with ascorbic acid plus α-tocopherol was not successful in the premature baboon model of prolonged hyperoxic injury. While antioxidant vitamin levels were successfully increased in animals treated with high-dose supplementation, no benefit was observed in terms of markers of lipid peroxidation, respiratory parameters, and lung histopathology [67]. Currently, the scientific data do not clarify whether the highly successful antioxidative approaches in rodents can be transferred to humans. Some concerns need to be kept in mind when referring to the field of MSC research, where highly successful strategies have so far not been recapitulated in rodents and humans [68]. These disappointing results, among, others have stimulated the search for downstream targets of ROS and peroxidation that might prove superior. Caffeine is currently highlighted in the clinical scenario for its efficacy in the respiratory drive, and its antioxidant capacity and lung-protective effects have been studied in preclinical newborn rodent models of hyperoxia-induced lung injury. Its action is related to reductions in glutathione, HO-1, and H_2_O_2_ levels, along with lipid peroxidation, and to a prevention of Nrf2 upregulation, while Keap1 levels and superoxide dismutases were preserved [69,70]. Within more detailed investigations, a reduction in oxidative stress was documented for the decrease in adenosine 2A receptor expression, reducing cell death induction in the lung—especially alveolar epithelial type II cells—NLRP3 inflammasome protein expression, and NF-κB pathway activation [70]. The multiple actions of caffeine were further unraveled when caffeine treatment during hyperoxia in newborn rats attenuated cyclooxygenase-2 activation and endoplasmic reticulum stress—a further downstream target of ROS beyond inflammation [71]. Selective inhibition of cyclooxygenase-2 (COX2) in a newborn rat model of hyperoxia exposure provided comparable results, with the exception that the changes in alveolar diameter were not abrogated, despite the preservation of the number of lung mesenchymal stem cells [72]. Studies in COX2-knockout mice and the application of COX2-specific inhibitors in newborn mice further indicated that COX2 is crucial for pro-inflammatory cytokine production and the influx of inflammatory cells in the lungs, but its inhibition was not effective in preventing hyperoxia-induced histopathological changes in mice exposed to 85% oxygen for 14 days, constituting a severe lung injury [73].

In addition to the prevention of hyperoxia-mediated ROS production and lung injury, the consequences of intermittent hypoxemic episodes have become the focus of more and more research, as stated in the introduction. Treatment with a peroxynitrite decomposition catalyst that catalyzes the isomerization of peroxynitrite (ONOO^−^)—a highly reactive oxidant formed by the combination of nitric oxide and superoxide anions during the phases of intermittent hypoxemia after hyperoxic exposure—reduced pulmonary inflammation and the secondary injuries to lung structures provoked by the double hit, particularly affecting lung and pulmonary vascular function [74]. These data confirm that secondary injurious insults during the recovery phase of the lungs after hyperoxia are of major importance, and that the repetitive desaturations that are frequently observed in preterm infants during the phase of stabilization are of high importance to further lung development, although these mechanistic data are so far restricted to rodent models. 

### 4.3. Anti-Inflammatory Drugs

Prenatal and postnatal corticosteroids constitute one of the cornerstones of preventing or treating BPD. Recently, postnatal topical corticosteroid application in the lungs in conjunction with a surfactant came into the focus of research, based on one clinical trial demonstrating its superiority. This combination of surfactant plus budesonide was recently studied in a model of mechanically ventilated preterm lambs. It is of particular importance that this strategy not only reduced the pulmonary inflammatory response and lung injury, but also attenuated the systemic inflammatory response in the brain and liver [75]. Due to the serious side effects on psychomotor and endocrine function, more selective therapeutic approaches are being extensively studied. It should be mentioned that natural surfactant preparations contain SOD and CAT activity, exerting antioxidative properties [54,76]. Molecular in vitro studies indicate that dipalmitoylphosphatidylcholine from surfactant preparations has anti-inflammatory properties, inhibiting the ATP-induced inflammasome activation and maturation of IL-1β via a mechanism involving nicotinic acetylcholine receptors [77]. The most encouraging approaches in preclinical studies target the IL-1β activation, including the interleukin-1 receptor antagonists (IL-1Rα), or modulators of the NOD-, LRR- and pyrin domain-containing 3 (NLRP3) inflammasome upstream of IL-1β [78]. These data were further confirmed in independent studies where genetic knockout of NLRP3 prevented caspase-1 activation, IL-1β, and lung inflammation [79]. Similarly, treatment with a Rac1-specific inhibitor that controls the NLRP3 inflammasome-mediated processing of pro-IL-1β into its active mature form markedly decreased the inflammasome activation and macrophage recruitment into the lungs [80]. One further strategy aimed to preserve heat shock protein 70 (Hsp70) expression via geranylgeranylacetone, which exerts a cytoprotective function via several anti-apoptotic and anti-inflammatory mechanisms, including the attenuation of ROS-mediated lipid peroxidation. In these animals, geranylgeranylacetone prevented the hyperoxia-mediated downregulation of Hsp70, and suppressed lung cell apoptosis induction and lung structural changes [81].

## 5. Outlook and Perspectives

Preterm birth is associated with fundamental changes in oxygenation. Therefore, the term “oxidative diseases” of the preterm infant perfectly characterizes the postnatal situation [10]. This can be particularly applied to the immature lung and the evolution of BPD. The past decade has been characterized by tremendous gains in knowledge of the pathomechanisms of BPD in the context of ROS, although their fundamental impact on further lung development has been acknowledged for decades. Targeting the critical pathways of ROS action proved effective in rodents exposed to hyperoxia. Despite advanced knowledge in preclinical studies, thus far, none of these therapeutic approaches has been successfully translated into clinical routine. Of course, one must acknowledge the proven efficacy of caffeine [68]. It will, however, be difficult to separate its benefits in terms of stabilizing the respiratory drive of the preterm infant and its antioxidant properties in the clinical setting, since distinguishing these effects is not within the focus of neonatologists. Rather, optimization of the dosing of caffeine in the context of prevention of mechanical ventilation, limitations in lung function, and psychomotor outcomes will guide future trials, as is currently the aim for vitamin A [82,83]. For vitamin A and vitamin D, the data do not allow a final conclusion thus far. It will not be easy to specify antioxidative effects of specific therapies within the complexity of BPD’s evolution [53,68]. The preclinical models have their limitations, which can be traced back to differences in the evolutionary status of the lungs compared to the preterm infant, along with the fact that the studies were exclusively performed for isolated injurious insults, while the pathogenesis of BPD in preterm infants is highly complex [84,85]. In order to specify examples showing that the preclinical progress is suited to shape future research, directions are based on the fact that ROS cause comparable downstream events in rodents and preterm infants, and lead to comparable persistent epigenetic changes. Further incorporating the insights and knowledge from these models into the clinical context will pave the way towards targeted therapies. This might be particularly applicable to therapies to prevent ROS injury, as multiple factors contributing to BPD aggravate ROS production, which has been most studied for oxygen therapy and bacterial infections. The same might account for microbial colonization of the lungs, the action of microbial axes, and the impact of nutrition. Taking into account that the downstream effects of ROS—including modification of the gene methylation status or apoptosis processes—cannot be classified as safe, the clinical approaches are still limited to dampening the inflammatory response, which is highly reliant on the high potency and broad anti-inflammatory activity of corticosteroids [54,68]. One might speculate that BPD, as a complex disease with multiple origins, cannot be tackled with one specific therapy. Novel approaches—including the application of allogeneic MSCs, with their broad anti-inflammatory and growth-promoting effects—are highly promising. Whether they will meet with equal success as in the rodent models requires scientific confirmation [68].

Today, it is much too early for a final assessment of whether ROS targeting is ineffective in preventing BPD, taking into account the high impact of ROS on lung development and the tremendous impact of small variations in oxygen targeting in the preterm infant. Rather, it remains a highly attractive area of research, and we will shed light on the clinical aspects and needs in the second chapter of our review [54].
